# Evidence to Use Botulinum Toxin Injections in Tension-Type Headache Management: A Systematic Review

**DOI:** 10.3390/toxins9110370

**Published:** 2017-11-15

**Authors:** Mieszko Wieckiewicz, Natalia Grychowska, Marek Zietek, Gniewko Wieckiewicz, Joanna Smardz

**Affiliations:** 1 Department of Experimental Dentistry, Wroclaw Medical University, 50-425 Wroclaw, Poland; joannasmardz1@gmail.com; 2 Department of Prosthetic Dentistry, Wroclaw Medical University, 50-425 Wroclaw, Poland; natgrychowska@gmail.com; 3 Department of Periodontology, Wroclaw Medical University, 50-425 Wroclaw, Poland; marekzietek@gazeta.pl; 4 City Hospital No. 4, 44-100 Gliwice, Poland; gniewkowieckiewicz@gmail.com

**Keywords:** tension type headache, botulinum toxin, injections

## Abstract

Tension-type headache (TTH) is the most common type of chronic recurring head pain. It can occur twice as often in women as in men. It is the most common type of headache. Its lifetime prevalence is 30% to 78% in the general population. TTH treatment should be multilevel. It often consists of taking pain medication, muscle relaxants, antidepressants, using biofeedback therapy, acupuncture, and attending behavioral therapy. Several clinical trials also suggest that botulinum toxin (BTX) may be an effective treatment option for such patients. The aim of this study was to evaluate if BTX can be used as a treatment method in TTH in the light of current medical literature. The authors searched the PubMed, EBSCOhost, OVID, Web of Knowledge, Cochrane Library and CINAHL databases to identify relevant publications. The authors finally included 11 papers—prospective and retrospective cohort studies. Among most of the selected studies, there was a significant correlation between using BTX and reduction of TTH pain intensity and severity. By analyzing qualified studies, it can be concluded that botulinum toxin seems to be effective in TTH management.

## 1. Introduction

A tension-type headache (TTH) is the most common type of chronic recurring head pain. It can occur twice as frequently in women as in men. It is the most common type of headache. Its lifetime prevalence is 30% to 78% in the general population. The International Headache Society (IHS) classification in 2013 includes TTH and categorizes it as episodic or chronic; it is also defined by whether it is associated with pericranial tenderness. The majority of people who suffer from TTH have episodic headaches, which appear one or two times per month on average. However, tension headaches can also be chronic [1,2,3,4]. A chronic tension-type headache (CTTH) is defined as the occurrence of tension-type headaches at a frequency of ≥15 days per month and it usually evolves from the episodic form. It causes significant functional impairment [5]. Both muscular and psychogenic factors are believed to be associated with tension-type headache. TTH can be caused by contraction of muscles in regions of the head and neck. It can be triggered by using a computer screen, long driving duration, eye strain, dry eyes, fatigue, smoking, alcohol, caffeine, emotional stress or even cold temperatures. Main symptoms of TTH are pressure around the forehead, dull head pain and tenderness around the forehead and/or scalp. It can appear as mild, moderate, or intense pain in the head, neck and/or behind the eyes. TTH is often defined by patients as a feeling of having a tight band around their forehead [6]. TTH has a very high socio-economic impact. This is why TTH treatment should be multilevel. It often consists of taking pain medications, muscle relaxants, antidepressants, using biofeedback therapy, acupuncture, and attending behavioral therapy. Several clinical trials also suggest that botulinum toxin (BTX) may be an effective treatment option for such patients.

Botulinum toxin (BTX) is a neurotoxic protein produced by the bacterium Clostridium botulinum and related species. BTX prevents the release of the acetylcholine neurotransmitter from axon endings at the neuromuscular junction. This action causes muscle paralysis [7]. There are eight types of BTX, A–H. Types A and B are often used, both commercially and medically [8]. In TTH treatment, botulinum toxin type A (BTX-A) is used. Its therapeutic effect in treating headaches was first noticed by Wiliam Binder in 1992 [9]. He reported an associated decrease in migraine recurrence after cosmetic injection of BTX-A to reduce forehead wrinkles. The working mechanism BTX-A affects the neuronal signaling pathways activated during a headache. It also has a blocking action on the parasympathetic nervous system and might inhibit the release of other neurotransmitters or affect the transmission of afferent neuronal impulses [10].

The aim of this study was to determine whether BTX can be used as a treatment method in TTH management in the light of current medical literature.

## 2. Results

### 2.1. Description of Studies

**Figure 1:**
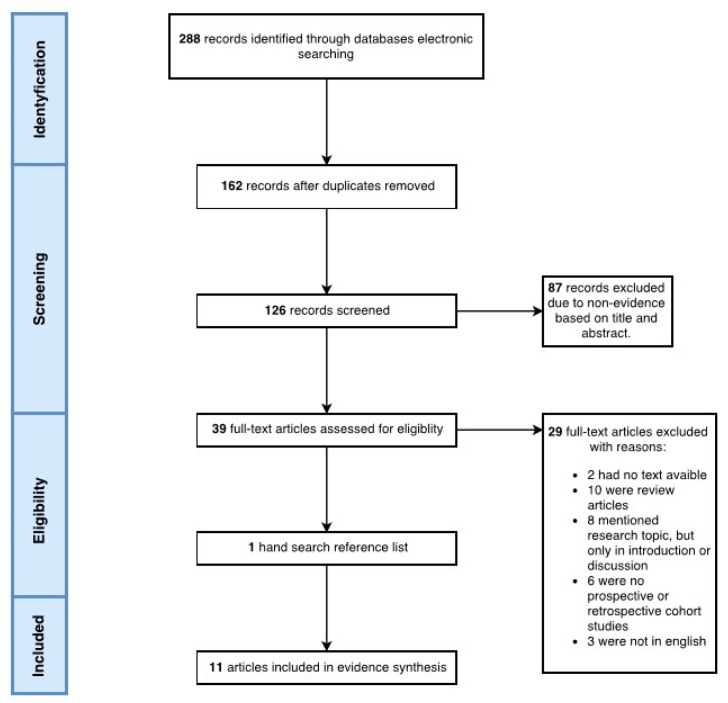
Flow diagram of the systematic review protocol.

The authors obtained 288 results from January 2007 to August 2017 in accordance with the research protocol. Eleven articles were included. The main exclusion reasons were the design of the study and the research topic mentioned only in the introduction. The protocol of the systematic review is presented as a flow diagram in Figure 1. Most studies have found that BTX can be very useful in TTH treatment [11,12,13,14,15,16,17,18,19,20]. There were also studies that did not support this thesis [21]. A fact that the problem of TTH is associated mainly with the female gender is an interesting issue to mention [11,12,13,15,17,18,19,21].

### 2.2. Characteristics of the Subjects Included in the Primary Studies

The total number of participants included in the studies ranged from 125 to 5. Subjects were adults, mainly females in most studies. In one study, the sample was composed only of children [10]. In most participants, TTH or CTTH was found [11,12,13,14,15,17,18,20,21]. In two studies, researchers also qualified patients with migraine [16,19]. In one study, participants without TTH also had masseter muscle pain related to temporomandibular joint dysfunction [14].

### 2.3. Quality Assessment

The authors finally included 11 papers—prospective and retrospective cohort studies. They also reported this kind of study the most reliable.

### 2.4. Synthesis of Evidence

Table 1 presents the quality of the evidence as an overall GRADE score for the primary outcome. The initial GRADE score of included studies was decreased due to the study design. Other common causes of reduced scores were clinical heterogeneity between studies and indirectness.

#### Primary Outcome

Among selected studies, in 10 there was a significant correlation between using BTX and reduction of TTH pain intensity and severity [11,12,13,14,15,16,17,18,19,20]. The quality of evidence in this group was moderate. In one study, headache intensity over time did not differ significantly when compared to the control group [21]. The quality of evidence in this study was also moderate.

## 3. Discussion

Information on sample size, type of injected BTX, dose, injected trigger points and the main reported outcomes are presented in Table 2.

Most studies reported a significant correlation between using BTX and a reduction in TTH pain intensity and severity [11,12,13,14,15,16,17,18,19,20]. Among described studies, some researchers proposed TTH treatment based on one injection session [11,12,13,14,15,18,19]. De Ru et al. conducted a study in a group of 10 (mostly females) patients suffering from frontal localized TTH using one session of BTX-A injections and reported that all patients had less pain for approximately two months after injection. Moreover, this kind of treatment did not appear to have lasting side effects. In another study conducted in 2011, scientists reported similar findings [11,12]. In both studies, place of injection was the corrugator supercilis muscle. Erdemoglu et al. conducted a study of which the aim was to investigate the long-term efficacy and safety of BTX-A for refractory CTTH. A total of 28 patients (mostly females) took part in the study and received one session of BTX-A injections into trigger points located within the frontalis, splenius capitis, trapezius, occipitalis and temporalis muscles. Researchers reported a reduction in pain frequency and intensity over 1 year. Pihut et al. reported similar findings in a study conducted in a group of 42 patents with masseter muscle pain related to temporomandibular joint dysfunction and tension-type headache. After injecting BTX-A into the masseter muscles, a number of referred pain episodes and pain in the temporal region decreased significantly. It was the only study in which masseter muscles were injected with BTX and which reported effectiveness of BTX both in TTH and masseter muscle pain treatment [14].

In some studies, scientists proposed injecting trigger points with BTX in more than one session [6,7,10]. Dowson et al. conducted a study in a group of 24 (mostly females) patients who were given three sets of BTX-A injections at 8- to 12-week intervals. They reported significant improvements in headache-related disability, pain and emotional function, headache frequency and lower medication use after treatment with BTX-A. Mathew et al. tried to evaluate predictions of response to BTX in patients with chronic daily headache. This researcher examined 71 patients with chronic migraines and 11 patients with CTTH. Patients received at least 2 sets of injections at intervals of 12–15 weeks. A greater percentage of patients witch chronic migraine responded to BTX-A than patients with CTTH, although, it was effective under both conditions. It was one of two studies in which participants were also patients with migraines [16,19]. Dowson et al. conducted a study in a group of 24 patients (mostly females) who were given three sets of BTX-A injections at 8- to 12-week intervals. They also reported significant improvements in headache-related disability, pain and emotional function, headache frequency and medication use after treatment with BTX-A [17].

Some research teams proposed conducting a study by dividing participants into subgroups taking into account the type of headache [16,19]. Venancio et al. conducted a study in a group of 45 (mostly females) patients suffering from myofascial pain and headaches. Among selected patients, 25% of them presented with tension-type headache, 15% with migraine and 60% with mixed headache (mainly TTH). The patients were divided into three groups: (1) dry-needling as a control group, (2) lidocaine injection, (3) botulinum toxin injection. Each patient was injected in one to three trigger points located based on headache complaint. Researchers proved that tested substances had desirable effects on the studied disorders, although, BTX should be reserved for cases in which effects could not be achieved using other methods [19].

There were also studies taking into account a comparison with a control group injected with a placebo [8,11]. Hamdy et al. reported a significant improvement after 1 month of BTX-A injection regarding headache insensitivity after conducting a study in a group of 28 Egyptian patients (mostly females). They were allocated into two groups; one received 100 U of BTX-A, and the other group received a placebo. Injected tender points were identified from potential tender points in the frontalis, temporalis, sternocleidomastoideus, trapezius, splenius capitis, and semispinalis muscles [18].

In most studies, participants were adults [11,12,13,14,15,16,17,18,19,21]. There was only one study in which participants were children. Schroeder et al. described the treatment of 5 children using the “StiBio” approach, consisting of an individualized headache intervention program. It consisted of analgesics, muscle relaxation, bio-behavioral management, pharmacological therapies and muscular trigger point therapy. BTX-A injections were given 1–4 times. In the long-term follow-up, chronic headache did not exist in any of the patients. This is also the only study in which the research team proposed a multilevel system of treatment.

In all studies, scientists used BTX-A. In most of them, Botox (Allergan) was injected [11,13,14,16,17,18,20]. In one study, scientists used Dysport (Ipsen Beaufour). Straube et al. conducted a study in a group of 125 patients (mostly females) with TTH using Dysport BTX. Treatment with 420 U of BTX was associated with significantly improved global physician and patient assessment scores and decreased headache episodes. Scientists also reported that further studies should address the possible value of multiple injections with extended observation periods, dose optimization, and whether duration of headache history and number of previous treatments are predictors of patient response [15].

Only one study did not support the assertion between using BTX and reduction in TTH pain intensity and severity. Harden et al. conducted a study in a group of 32 participants (mostly females) with TTH. After injection of BTX-A, reduction of headache intensity over time did not differ significantly compared to the control group [21].

The most commonly injected muscles include the trapezius [13,15,18,20,21], corrugator [11,12,15,16], temporalis [13,15,16,18] and frontalis [13,15,16,18]. Every administration of BTX in confirmatory studies resulted in a decrease in TTH symptoms regardless of dose [11,12,13,14,15,16,17,18,19,20]. In studies where a higher dose or more muscles were injected, the effect was more rapid than in studies proposing lower doses and fewer injected muscles [13,15,16,17,18]. On the other hand, maintaining a therapeutic effect was more durable and easier in patients injected in a few cycles with intervals than in patients injected in one cycle [6,7,10].

## 4. Conclusions

By analyzing qualified studies, it can be concluded that BTX is useful in the treatment of TTH. However, the results of treatment are dependent on the dose, location of injection, number of cycles and intervals. Treatment outcomes may also be modified by accompanying illnesses such as temporomandibular disorders. Botulinum toxin seems to be effective in treating TTH, but it should be reserved for refractory cases, in which the expected effects cannot be achieved by using other methods. The problem should therefore be better investigated considering a larger group of patients.

## 5. Materials and Methods

The Preferred Reporting Items for Systematic Review and Meta-Analysis (PRISMA) guidelines were followed by the authors for this systematic review for the collection and reporting of data [22,23].

### 5.1. Eligibility Criteria for Initial Study Selection

#### 5.1.1. Studies

The authors used the presented inclusion criteria: retrospective and prospective cohort studies that discussed the treatment of TTH with BTX. English language and full text peer-reviewed articles published after 1 January 2007 were included in the study.

#### 5.1.2. Participants

Participants were males and females of any age with clinical diagnosis of TTH.

#### 5.1.3. Outcomes

A study was included in this systematic review if it investigated the use of BTX in TTH treatment.

### 5.2. Data Sources and Searches

The authors searched the PubMed, EBSCOhost, OVID, Web of Knowledge, Cochrane Library and CINAHL databases to identify relevant publications. The authors added filters to identify prospective and retrospective cohort studies and to ensure the searching process was accurate. Additional filters included articles published after 1 January 2007 and those available in English. The literature search strategy was based on Medical subject headings (MeSH) [22,23] as follows: each of two synonymous phrases, i.e., (1) tension type headache, (2) tension-type headache, were combined with each of: (a) botulinum toxin, (b) botulinum toxins, e.g., “tension type headache botulinum toxin,” viz. (1) + (a); “tension type headache botulinum toxins,” viz. (1) + (b), etc. In this way, 4 queries were obtained. The authors scoured the reference list of included studies to identify other potentially appropriate studies.

### 5.3. Trial Selection

In the literature screening procedure, all authors were involved. Firstly, authors screened the titles, abstracts and full texts. Then, the authors screened the full texts for key words, such as “botulinum,” “toxin,” “tension,” “headache,” to find those that were potentially suitable. Then, they finally evaluated studies of practical validity regarding the use of BTX in TTH treatment. Finally, the authors decided together if all of the chosen articles fulfilled the inclusion criteria. None of the review authors was blind to the journal title or to the study authors or institutions [23].

### 5.4. Data Extraction

The data extraction process focused on information about sample size and characteristics, diagnosis, use of BTX, dose, location of injection and outcomes.

### 5.5. Data Synthesis and Analysis

The authors conducted a narrative, qualitative summary. The Grading of Recommendations Assessment, Development and Evaluation working group approach was used to assess the quality of evidence [24]. One of the following categories: very low, low, moderate or high, was used to assess the quality of evidence for each main outcome.

## Figures and Tables

**Table 1:** Summary findings for the primary outcomes.

Primary Outcome	Outcome Significance	Trials (Year)	No. of Participants (Studies)	Quality of the Evidence (Grade)
Decreased TTH	Significant correlation	De Ru et al. (2011) [11]	233 (ten studies)	+++− moderate due to indirectness
		De Ru et al. (2008) [12]		
		Erdemoglu et al. (2007) [13]		
		Pihut et al. (2016) [14]		
		Straube et al. (2008) [15]		
		Mathew et al. (2007) [16]		
		Dowson et al. (2008) [17]		
		Hamdy et al. (2008) [18]		
		Venancio et al. (2009) [19]		
		Schroeder et al. (2012) [20]		
	No significant correlation	Harden et al. (2008) [21]	32 (one study)	+++− moderate due to indirectness

Legend for the Quality of Evidence: high (++++); moderate (+++−); low (++−−); very low (+−−−).

**Table 2:** Information for selected studies.

Author (Year)	Sample Size	Botulinum Toxin	Total Dose Per Session	Injection Place	Main Outcome
De Ru et al. (2011) [11]	10	BTX-A (Botox, Allergan)	25 U	corrugator supercilii muscle	90% of patients had a drastically lowered pain score post-operatively
De Ru et al. (2008) [12]	10	BTX-A	25 U	corrugator supercilii muscle	All patients had less pain for approximately two months
Erdemoglu et al. (2007) [13]	28	BTX-A (Botox, Allergan)	45–75 U	frontalis, splenius capitis, trapezius, occipitalis, temporalis muscle	Headache frequency was found to be significantly reduced
Pihut et al. (2016) [14]	42	BTX-A (Botox, Allergan)	42 U	masseter muscle	A decrease in the number of referred pain episodes and a decrease in pain in the temporal region
Straube et al. (2008) [15]	125	BTX-A (Dysport, Ipsen Beaufour)	210–420 U	trapezius, splenius, temporalis, frontalis, corrugator muscles	Decreased headache episode number in a group receiving 420 U of BTX-A
Mathew et al. (2007) [16]	82	BTX-A (Botox, Allergan)	100 U	procerus, corrugator, frontalis, temporalis, occipitalis, suboccipitalis muscles	A greater percentage of patients with chronic migraine responded to botox than patients with CTTH
Dowson et al. (2008) [17]	24	BTX-A (Botox, Allergan)	30–100 U	bilaterally at the site of the first trigeminal nerve and the rest of the dose to follow the pain in the cervical area	Significant improvements in headache-related disability, pain and emotional function, headache frequency and medication use
Hamdy et al. (2008) [18]	28	BTX-A (Botox, Allergan)	100 U	frontalis, temporalis, sternocleidomastoideus, trapezius, splenius capitis, semispinalis muscles	Significant improvement after 1 month of BTX-A injection regarding headache days/month
Venancio et al. (2009) [19]	45	BTX-A	25 or 50 U	trigger points	Significant improvement after injection regarding headache days/month
Schroeder et al. (2012) [20]	5 (children)	BTX-A (Botox, Allergan)	20–90 U	splenius, trapezius, semispinalis, scalenius muscles	In the long-term follow up, headache did not exist in any of patients
Harden et al. (2008) [21]	32	BTX-A	25–100 U	trapezius, sternocleidomastoid, splenius capitis muscles	Reduction of headache intensity over time did not differ significantly when compared to the control group
